# Cardiac Biomarkers and Autoantibodies in Endurance Athletes: Potential Similarities with Arrhythmogenic Cardiomyopathy Pathogenic Mechanisms

**DOI:** 10.3390/ijms22126500

**Published:** 2021-06-17

**Authors:** Ilaria Stadiotti, Melania Lippi, Angela Serena Maione, Paolo Compagnucci, Daniele Andreini, Michela Casella, Giulio Pompilio, Elena Sommariva

**Affiliations:** 1 Unit of Vascular Biology and Regenerative Medicine, Centro Cardiologico Monzino IRCCS, 20138 Milan, Italy; mlippi@ccfm.it (M.L.); amaione@ccfm.it (A.S.M.); gpompilio@ccfm.it (G.P.); esommariva@ccfm.it (E.S.); 2 Cardiology and Arrhythmology Clinic, University Hospital Ospedali Riuniti Umberto I-Lancisi-Salesi, 60126 Ancona, Italy; paolocompagnucci1@gmail.com (P.C.); michelacasella@hotmail.com (M.C.); 3 Unit of Cardiovascular Imaging, Centro Cardiologico Monzino IRCCS, 20138 Milan, Italy; dandreini@ccfm.it; 4 Department of Clinical Sciences and Community Health, Università degli Studi di Milano, 20122 Milan, Italy; 5 Heart Rhythm Center, Centro Cardiologico Monzino IRCCS, 20138 Milan, Italy; 6 Department of Clinical, Special and Dental Sciences, Marche Polytechnic University, 60126 Ancona, Italy; 7 Department of Biomedical, Surgical and Dental Sciences, Università degli Studi di Milano, 20122 Milan, Italy

**Keywords:** arrhythmogenic cardiomyopathy, athletes, autoantibodies, physical exercise, desmosomes

## Abstract

The “Extreme Exercise Hypothesis” states that when individuals perform training beyond the ideal exercise dose, a decline in the beneficial effects of physical activity occurs. This is due to significant changes in myocardial structure and function, such as hemodynamic alterations, cardiac chamber enlargement and hypertrophy, myocardial inflammation, oxidative stress, fibrosis, and conduction changes. In addition, an increased amount of circulating biomarkers of exercise-induced damage has been reported. Although these changes are often reversible, long-lasting cardiac damage may develop after years of intense physical exercise. Since several features of the athlete’s heart overlap with arrhythmogenic cardiomyopathy (ACM), the syndrome of “exercise-induced ACM” has been postulated. Thus, the distinction between ACM and the athlete’s heart may be challenging. Recently, an autoimmune mechanism has been discovered in ACM patients linked to their characteristic junctional impairment. Since cardiac junctions are similarly impaired by intense physical activity due to the strong myocardial stretching, we propose in the present work the novel hypothesis of an autoimmune response in endurance athletes. This investigation may deepen the knowledge about the pathological remodeling and relative activated mechanisms induced by intense endurance exercise, potentially improving the early recognition of whom is actually at risk.

## 1. Introduction

Although physical exercise is recommended for the maintenance of a healthy lifestyle and the reduction of cardiovascular disease incidence [1,2], prolonged and intense activity can be deleterious for cardiac structure and function. It can acutely and transiently increase sudden cardiac death (SCD) and myocardial infarction risk in susceptible individuals [3]. Increased myocardial fibrosis [4,5], coronary artery calcification [6], and atrial fibrillation [7,8] have been reported in endurance athletes.

**Figure 1:**
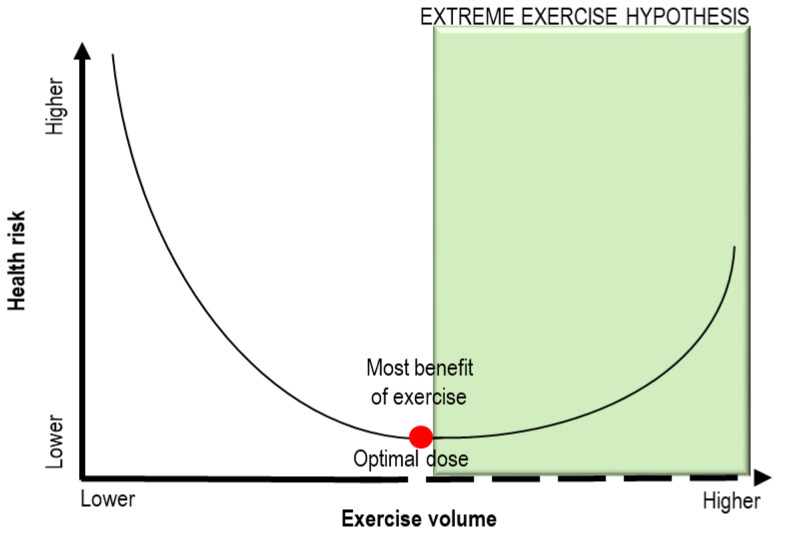
Schematic representation of the “Extreme Exercise Hypothesis”. A J-shaped (or U-shaped) curve describes the dose –response relationship between exercise training volumes and health risk. When the threshold of optimal exercise dose (red point) is exceeded, the health benefits of training can be reduced. Adapted from [9].

Since endurance athletes exceed the usual recommendations for exercise by 15-fold to 20-fold, the “Extreme Exercise Hypothesis” has been proposed to explain how the beneficial effects of physical activity may plateau or decline when individuals perform training beyond the ideal exercise dose [9,10]. As depicted in Figure 1, the dose–response relationship between exercise training volumes and health risk is described by a J-shaped (or U-shaped) curve [2,9]. To date, the exact amount of exercise able to impair the cardiovascular system has not been defined. The metabolic equivalents of task (METs) method is recognized as useful to evaluate the functional capacity or exercise tolerance of an individual [11]. One MET is the amount of oxygen consumed at rest and is equal to 3.5 mL of oxygen per kilogram per minute [11]. Most reports have defined “vigorous exercise” as needing at least six METs, although the maximal individual capacity could influence this threshold [3].

Most exercise-associated adverse effects often occur in subjects with occult or diagnosed structural cardiac diseases [3,12]. Among young individuals, the concomitant pathological conditions are often hereditary or congenital cardiovascular abnormalities, such as arrhythmogenic cardiomyopathy, hypertrophic cardiomyopathy, coronary artery anomalies, and bicuspid aortic valve [13,14,15]. Among older subjects who die during physical exercise, coronary artery disease is the most frequent pathological finding [16,17].

SCD overall incidence during exercise is estimated at 1:50,000 [18,19,20]. Exercise-related SCD seems to depend on the interaction between the physical activity acute trigger and an underlying disease, but it can be further elicited by other concomitant processes, including emotional stress, hemodynamic changes, and impaired parasympathetic tone [3]. It has been reported that physical activity may increase the risk of SCD by 2.5 times [21].

In the present review, we summarize the current knowledge about exercise-induced cardiac alterations and circulating biomarkers of damage. In addition, we propose a novel hypothesis of an autoimmune response in endurance athletes, based on the analogies with arrhythmogenic cardiomyopathy patients.

### 1.1. The Athlete’s Heart

Vigorous physical exercise is associated with significant changes in myocardial structure and function. The athlete’s heart has to sustain a higher cardiac output during maximal effort than untrained hearts. Thus, it is subjected to a physiological remodeling that allows its greater resistance during intense activity and sufficient oxygen delivery to exercising muscles. This physiological response is known as the Frank–Starling Mechanism or “law of the heart” [22].

Sympathetic activation is responsible for the augmented cardiac output through heart rate modulation. The heart rate spans from <40 bpm at rest to >200 bpm in a young maximally exercising individual. The stroke volume may significantly increase with sustained training because of the higher ventricular end-diastolic volume and sympathetically mediated end-systolic volume reduction [23]. The hemodynamic changes parallel cardiac chamber enlargement and hypertrophy [24,25,26]. These cardiac adaptations may mimic those of a diseased heart, but in most cases systolic and diastolic functions are preserved [27,28], although a transient reduction in left ventricular (LV) ejection fraction (EF) has been reported after more than 6 h of continuous exercise [29,30]. In many cases, the right ventricular (RV) function seems to be more compromised by prolonged exercise than left ventricular one, possibly for the thinner-walled structure of RV [31,32,33]. Indeed, RV wall stress increases more than in the LV during exercise, producing higher pressure load on the RV, not compensated by a sufficient volume increase and myocardial thickening [34]. This depends on the increase in pulmonary artery pressure relative to systemic vascular pressure, necessary to guarantee the requested cardiac output [35]. However, at equal exercise loads, the RV response shows high interindividual variability due to differential adaptations of the pulmonary circulation. Indeed, a higher vascular reserve corresponds to enhanced maximal exercise capacity [36]. In addition, conduction alterations are common, and they are usually mediated by parasympathetic activity and/or sinoatrial node slowing [37]. Endurance athletes are often affected by bradyarrhythmias, such as sinus bradycardia, junctional bradycardia, and first-degree atrioventricular block [38]. Trained athletes can experience premature beats and non-sustained ventricular tachycardia, usually of benign etiology and without long-term consequences [39,40], although no higher prevalence if compared to sedentary individuals has been assessed [41]. The main problematic effect of intense physical activity is atrial fibrillation, which has been reported more frequently among athletes than in sedentary individuals [42,43]. Syncope often manifests in the immediate post-exercise period due to neurocardiogenic mechanisms based on a sudden reduction in venous return. When syncope manifests during exercise, it can be due to malignant arrhythmias, structural cardiac disease, or myocardial ischemia, which have to be thoroughly evaluated [44].

Although these alterations are often reversible, long-lasting cardiac damage may develop after years of intense physical exercise [45,46].

Furthermore, after prolonged endurance exercise, myocardial inflammation, oxidative stress, and fibrosis have often been reported, representing a substrate for life-threatening arrhythmias [5,10,47].

During exercise, increased metabolic processes with an augmented oxygen uptake may induce a mitochondrial electron “leakage” and the consequent production of reactive oxygen species (ROS) [48,49]. Moreover, the activation of immune and inflammatory responses due to exercise-induced muscle injury may generate high amounts of ROS [50,51]. To possibly counterbalance the oxidative damage, an increase in antioxidant defenses, through the activation of antioxidant enzymes [52], has been reported in response to high volumes of exercise [48]. Thus, it seems that oxidative stress does not occur below a certain threshold of intensity, but only when exercise is strenuous [53].

The higher amount of oxidative stress can increase oxidation of different molecules, causing their damage. For example, acute bouts of exercise can increase LDL oxidation [54,55,56,57].

The oxidative radicals could impair cardiomyocyte membrane permeability, concurring with mechanical stress in cardiomyocyte remodeling [58,59]. In addition, ROS may inhibit glycolysis, producing a perturbation in calcium homeostasis, which could lead to myocardial dysfunction [60].

Exercise-induced myocardial fibrosis patterns are various and differ according to the age of the athletes [61,62,63]. Fibrosis is often found near the interventricular septum, especially in middle-aged and older athletes, and near the right ventricular insertion points, mainly in young athletes [4,5,61]. More rarely, a sub-endocardial ischemic pattern, a sub-epicardial pattern, and extensive mid-wall and diffuse fibrosis could be detected. Since only specific fibrotic patterns have been associated with ventricular arrhythmias and adverse cardiac events, the clinical and prognostic significance of myocardial fibrosis in athletes is yet to be determined [63]. The dose of exercise has been reported to be associated with the extent of fibrotic substitution in few studies [5,9,64].

The impact of gender on the ventricular response to exercise constitutes a relevant open issue. Although male versus female differences have a strong impact on cardiovascular disease pathogenesis [65,66,67], female athletes are often underrepresented in studies of cardiac adaptation to exercise. Few studies on this topic demonstrated a similar cardiac remodeling and prevalence of arrhythmic events in both male and female athletes [68,69]. However, RV performance during exercise seems to be enhanced in women when compared to men [68]. Conversely, a recent study reported that LV remodeling is more common in males, whereas RV remodeling mainly concerns females [70]. Thus, further investigations are needed to clarify the effective impact of gender.

### 1.2. Effects of Myocardial Stretching

During intense exercise, the stretching of the myocardium activates different intrinsic physiologic mechanisms to adequately respond to this stimulus [71], through the so-called mechanoelectric feedback, which is able to transduce the mechanical stimulus into an electrical signal [72,73].

Each component of the heart seems to perceive mechanical stimuli, activating intracellular pathways that mediate several responses [74,75]. These pathways are often activated without binding of extracellular mediators [76]. An example is stretch-activated channels, able to modulate their permeability to ions and, consequently, electrical and mechanical properties of the myocardium [77]. This response often depends on protein phosphorylation. For example, calcium channel phosphorylation, by intensifying calcium transient, improves the contractile function [78]. Besides ion channels, other cardiomyocyte proteins concur in stretch-activated mechanisms: troponin I phosphorylation increases contractility, owing to the reduction of myofilament calcium sensitivity [79]; titin, both functioning as a mechanosensor and a molecular target, can trigger downstream signaling and modulate myocardial tension and sarcomeric length [80,81,82].

A central role in mechanosensing is played by intercalated discs, required to maintain mechanical and electric coupling between cardiomyocytes [83]. The two main structures of intercalated discs, fascia adherens junctions and desmosomes, contribute to adaptive responses to stretch [84] due to their connection with cytoskeletal actin and intermediate filaments, respectively [83]. In volume overload conditions, as during intense physical activity, the intercalated discs undergo dynamic changes [85]. For example, N-cadherin (N-CAD), one of the main proteins of fascia adherens junctions, is upregulated following mechanical stretch and elicits changes in cardiomyocyte shape, myofibrillar organization, and function [86]. On the contrary, N-CAD downregulation precludes the correct formation of intercalated discs, provoking cardiac morphological and functional defects [87].

Similarly, desmosomal protein loss impairs mechanotransduction responses [83]. For example, the deletion of the desmosomal protein desmoglein 2 (DSG2), necessary to assembly the extracellular domain, alters cell adhesion and signaling. It provokes the upregulation of heart failure markers, fibrosis, biventricular dilation and dysfunction, and death [88,89,90]. In general, desmosome deficiency leads to cardiomyocyte inability to appropriately face high mechanical stress, resulting in myocyte detachment and tissue remodeling [84].

Moreover, gap junctions, prominently localized at intercalated discs, mediate electrical propagation and are thus crucial to excitation and contraction [91]. They are composed of connexins, among which connexin 43 (CX43), the most important in the myocardium [91]. Physical exercise may affect gap junction remodeling, leading to CX43 expression downregulation during acute exercise, as demonstrated in a murine model [92], and to a possible consequent impairment of electrical conduction [93].

Additionally, costamere proteins, which are responsible for the connection between the contractile apparatus and extracellular matrix, as integrins, are involved in mechanotransduction and can be compromised when subjected to mechanical stress [94,95,96,97].

All these modifications generally have an adaptive meaning, but, depending on the strength of the stimulus, their nature, and the individual’s genetic background, they can result in maladaptive pathological remodeling [98], with mechanoelectric feedback dysfunction [72] and consequent arrhythmias, cardiac hypertrophy, and heart failure [74,84]. Indeed, when the physical activity is prolonged and intense, the impairment of these processes may provoke an altered cellular response and possibly heart disease.

### 1.3. Circulating Biomarkers of Exercise-Induced Damage

The changes induced by endurance exercise are associated with several circulating biomarker increases. These elevations are usually modest and transient, but their clinical implications are not fully elucidated.

As for cardiac damage biomarker, cardiac troponin (cTn) levels significantly increase after only 30 min of intense physical activity [99], reaching higher levels in younger and untrained individuals [100], concomitant with cardiovascular risk factors [101], greater exercise duration and intensity [32,101,102,103,104,105], and dehydration [106]. cTn release likely depends on exercise-induced cardiomyocyte necrosis, or the changes in membrane permeability caused by intense activity could determine the leakage of unbound troponin [107]. Further studies are needed to understand the mechanisms mediating its elevation [59,108]. Usually, cTn levels return to baseline within 72 h [109,110], and any cardiac dysfunction associated with increased cTn has been reported transient [59].

B-type natriuretic peptide (BNP) and its cleaved form NT-proBNP are secreted in response to cardiomyocyte stress produced by volume or pressure overload [111]. Thus, they can increase after endurance exercise [32,112,113,114,115,116,117], but return to baseline within 72 h [109,118]. Exercise duration [104,115], age [113,118], and poor physical preparation [32,112,119] can impact on BNP and NT-proBNP elevation.

Creatine kinase MB (CKMB), belonging to myocardial infarction biomarkers, can also be increased after intense activity, but it possibly originates more from skeletal muscle damage than from myocardial injury [120].

Moreover, typical fibrosis biomarkers have been associated with intense physical exercise. Soluble suppression of tumorigenicity 2 (sST-2) concentrations exceed the upper reference value after endurance activity, reaching higher levels as exercise intensity increases, but its complete normalization occurs within 48 h [121].

Tissue inhibitors of matrix metalloproteinase type I (TIMP-1), carboxy-terminal telopeptide of collagen type I (CITP), and carboxy-terminal propeptide of collagen type I (PICP) are other circulating markers of collagen synthesis and degradation that are augmented in endurance athletes [59].

Similarly, galectin-3 resting levels are greater in athletes than controls, and further increase after physical activity, possibly produced mainly by skeletal muscle [122]. Indeed, no correlations with cardiac function have been detected [122].

For what concern oxidative stress markers, 13- and 9-hydroxy-octadecadienoic acid (13-HODE and 9-HODE), known oxidized linoleic acid metabolites, significantly increase immediately post-exercise, but their plasma concentrations return to baseline levels within 24 h [123]. Their production could be linked to lipoxygenase activation in response to cell injury [124].

Moreover, lipid peroxidation increases after endurance exercise, as demonstrated by higher levels of malondialdehyde (MDA) [117,125] and F(2)-isoprostanes [57]. In both cases, the augmentation is transient.

Similarly, the heat shock proteins Hsp70 and Hsp72, known inflammation markers, are upregulated in athletes’ serum after physical exercise [126,127,128]. As for the majority of the exercise-induced circulating biomarkers, the increase is rapid but transient. Higher levels of these proteins has been previously reported in failing hearts [129]. Thus, their release is not specifically associated with exercise-induced alterations.

The current information about circulating factors in athletes is limited and a broader analysis (e.g., microRNA) is required [130,131]. To date, no biomarkers have been associated with the adverse remodeling that can occur after endurance activity with a cause–effect specificity. Indeed, most of the described biomarkers are elevated also in concomitance with various arrhythmic and heart failure diseases [132], making the understanding of the underlying causes difficult.

## 2. Exercise-Induced Arrhythmogenic Cardiomyopathy

Together with polymorphic ventricular tachycardia [133] and hypertrophic cardiomyopathy [134,135], arrhythmogenic cardiomyopathy (ACM) is included among the cardiac diseases that can be induced by physical exercise.

ACM is a genetic cardiac disorder, predominantly affecting the RV [136]. It is characterized by a fibro-adipose replacement of the ventricular myocardium, malignant arrhythmias, and SCD, especially in young adults and athletes [136]. Often, ACM penetrance is incomplete and genotype-phenotype correlations are difficult to establish [137]. Among the pathogenesis cofactors that have been proposed to explain ACM variable expressivity, physical exercise exposes ACM patients to a five-fold higher risk of SCD if compared to sedentary patients [138]. Due to the critical RV degeneration and arrhythmogenicity following repetitive intense exercise [31,32,33], even in the absence of known genetic abnormalities [139], the syndrome of “exercise-induced ACM” was proposed few years ago [140,141].

Although only a small fraction of high-level athletes develops signs of RV cardiomyopathy, the hypothesis is that endurance exercise provokes RV insults, in line with circulating cardiac damage biomarker rise and dysfunction, that, in the long term, could have pathological implications [142,143]. According to this hypothesis, an ACM-like phenotype may be acquired and not unquestionably ascribed to a genetic predisposition. Studies with a rat model of long-term intensive exercise training supported this theory, proving training-dependent RV fibrosis and increased arrhythmia inducibility after chronic endurance exercise [144].

If definite ACM patients carry, in the majority of the cases, genetic mutations that impair desmosomes, exercise-induced ACM is likely to involve cardiac junctions as well. Indeed, in the latter case, disproportionate wall stress, caused by intense and prolonged physical exercise, disrupts “normal” desmosomes [142], and the mismatch between wall stress and desmosomal integrity can be associated with the huge hemodynamic stress [142].

A debate about the effective existence of these exercise-induced ACM forms is still open and not all the involved factors have been unraveled. Indeed, no proper studies have confirmed or denied the existence of ACM phenotypes exclusively induced by exercise [145]. Additionally, gene-elusive patients represent a heterogeneous group, since some might have ACM causative genetic variants in genes not yet identified as associated. Possibly, other factors can predispose a subpopulation of endurance athletes to this condition, such as an unrecognized genetic predisposition that phenotypically manifests only in the setting of extreme exercise [146]. Moreover, the study of the underlying molecular mechanisms is still difficult for the limitations of the current in vivo models of endurance exercise [147].

**Figure 2:**
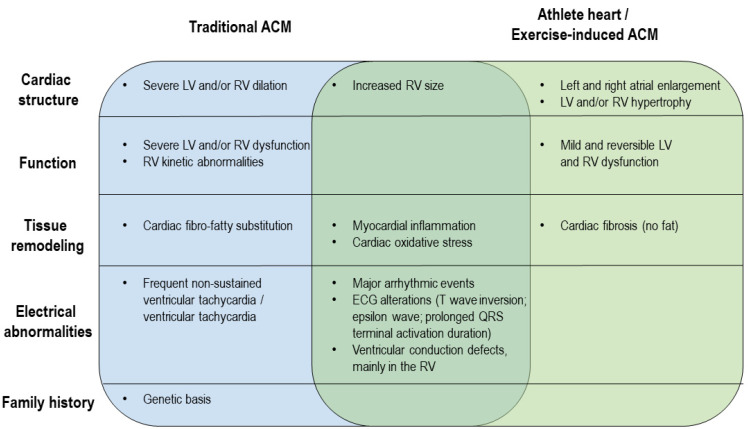
Comparison of ACM and exercise-induced ACM clinical phenotypes. The diagram illustrates differences and similarities between ACM and exercise-induced ACM from a clinical point of view.

The distinction between ACM and the athlete’s heart is still a challenge for sports cardiologists because of the overlapping features [148,149,150,151]. Indeed, studies comparing athletes with and without recognized genetic mutations often described similar clinical phenotypes (Figure 2). The proportion of individuals experiencing a major arrhythmic event during the follow up is comparable (28% of the cases), and electrocardiographic signs, including the ACM diagnostic major criterion epsilon wave, were reported to be similarly represented [139], except for the presence of pathological Q waves only in ACM patients [149]. The impairment of signal average ECG (SAECG) parameters has been more frequently reported in traditional ACM cases [149]. ACM patients are characterized by more severe LV and/or RV dilation and dysfunction, as confirmed by the stronger EF reduction [139,142], but the increase in RV size is similar in ACM and exercise-induced ACM individuals [152]. As for tissue remodeling, a typical feature of ACM patients is fibro-fatty substitution, whereas only fibrosis has been found in exercise-induced ACM individuals [139]. In addition, ACM patients exhibit delayed gadolinium enhancement [149]. On the other hand, histological abnormalities and cardiac inflammation and oxidative stress are shared by the two types of patients [12,142].

Unfortunately, despite the intensive efforts to discriminate traditional ACM patients from exercise-induced ACM individuals, there is still the need to improve the diagnostic procedure. In addition, the comparison between the features of ACM left dominant and biventricular forms to exercise-induced remodeling could be of further help.

## 3. Autoimmune Response Hypothesis in Endurance Athletes

Based on the available information, a parallelism between the negative effects induced by intense exercise at cardiac level and the typical characteristics of ACM, mainly caused by genetic mutations that affect desmosomes, has been proposed by coining the term “exercise-induced ACM” [140,141]. Although a proper description of the features that can discriminate the two forms is still lacking, some lessons might be learned from the actual knowledge of ACM. Recently, circulating autoantibodies against DSG2, one of the desmosomal proteins, have been found in ACM patients, and not in healthy controls and subjects affected by other cardiomyopathies [153]. These results are promising for the development of a novel diagnostic test for ACM patients and to potentially discriminate definite ACM patients from people affected by other conditions in differential diagnosis [132]. However, can they discriminate ACM from exercise-induced ACM?

The generation of these autoantibodies is likely to be dependent on DSG2 release into the intercellular space and circulation due to an ACM causative genetic mutation [153]. DSG2 may include “cryptic” epitopes that can induce, once exposed, an immune response [153].

However, no authors have to date assessed if the same autoimmune mechanisms interest endurance athletes. Indeed, as described above, among the induced modifications, intense physical exercise can challenge cell junctions, especially fascia adherens and desmosomes [84], and the expression and localization of the constituent proteins may be altered [85]. Although their remodeling usually ensues to properly respond to a greater mechanical stress, when a threshold of intensity is exceeded, dysfunction may occur due to increased oxidative stress [48,49] and changes in cell permeability [107]. The extent of fibrotic substitution that can follow endurance activity exacerbates the reduction of cell-to-cell contact [154]. Accordingly, the disruption of epithelial tight junctions has been already described during exercise due to heat- and oxidative damage-mediated stress [155]. Therefore, similar mechanisms are likely to impact on other cell junctions also at the cardiac level. In the worst scenario, all these processes could induce the detachment of intercellular junctional proteins, such as DSG2. For these reasons, the analysis of endurance athletes’ plasma for anti-DSG2 autoantibody is awaited to assess the potential of this immune biomarker in distinguishing ACM from athlete’s heart remodeling.

Other than their role as circulating biomarkers, DSG2 autoantibodies may affect cardiac function, as suggested by Chatterjee and colleagues [153]. Their levels correlate with premature ventricular contraction count in ACM patients, and in vitro analyses revealed their ability to functionally affect gap junctions. This is in line with the increasingly recognized ability of autoantibodies in arrhythmia stimulation through ion channels interference [156]. Moreover, DSG2 autoantibodies may attack the desmosomes and the whole intercalated discs, further weakening these structures. This is likely to happen similarly to what is described for anti-DSG3 autoantibodies produced against the cutaneous isoform of desmoglein in some skin disorders [157]. These autoantibodies induce DSG3 internalization and redistribution, altering the dynamics of desmosome assembly [158] and increasing tissue fragility in this already diseased state [159].

In addition, the inflammation triggered by this faulty immune response may aggravate the exercise-induced dysfunction [160]. 

In this view, in addition to physical detraining, immunosuppressive measures, including immunomodulatory drugs, plasmapheresis, or immunoadsorption for autoantibody removal, may be of help for at risk individuals.

## 4. Conclusions

Although physical exercise is an important measure to reduce cardiovascular disease incidence, excessive endurance training can paradoxically increase SCD and myocardial infarction risk in susceptible individuals as explained by the “Extreme Exercise Hypothesis”. Several exercise-induced features overlap with ACM and make the distinction between ACM and the athlete’s heart challenging. A debate about the existence of ACM phenotypes exclusively induced by exercise is still open. More information about the pathological remodeling and relative activated mechanisms induced by intense endurance exercise needs to be collected. Since not all the individuals practicing sports at high levels manifest exercise-induced cardiac alterations, the early recognition of whom is actually at risk is fundamental. The improvement of pre-participation screenings and the evaluation of new potential circulating biomarkers could be of considerable help.

## Data Availability

Not applicable.
